# Art-based interventions and social–emotional outcomes in children with autism spectrum disorder: a systematic review and meta-analysis with a proposed neurodevelopmental framework

**DOI:** 10.3389/fnhum.2026.1941559

**Published:** 2026-09-15

**Authors:** Liandi He, Rilei He, Guomeng Liu, Siying Liu, Xin He

**Affiliations:** 1 Guangdong Technician College of Light Industry, Guangzhou, China; 2 The First People's Hospital of Foshan, Foshan, China; 3 South China Normal University, Guangzhou, China; 4 Guangdong Shuangma Technology Co., Ltd., Guangzhou, China

**Keywords:** amygdala–prefrontal connectivity, art-based intervention, autism spectrum disorder, emotion regulation, emotional intelligence, frontoparietal network, social–emotional outcomes, systematic review and meta-analysis

## Abstract

Emotional intelligence (EI)-related social–emotional difficulties in children with autism spectrum disorder (ASD) are linked to atypical social-brain development, including altered amygdala-prefrontal, fusiform, and frontoparietal function during affective processing. This systematic review and meta-analysis quantified the effects of visual art-based interventions on EI and EI-related social–emotional outcomes in children with ASD, and integrated convergent neuroimaging evidence into a testable neurodevelopmental framework. Following PRISMA 2020, PubMed, Web of Science, PsycINFO, the Cochrane Library, ProQuest Dissertations, ClinicalTrials.gov, and gray literature were searched from inception to 31 December 2025, with an updated search on 15 May 2026 and a supplementary public-source update on 17 August 2026. Eligible studies included controlled, quasi-experimental, or pre-post visual art-based interventions lasting at least 4 weeks in children aged 3–18 years with clinically diagnosed ASD. Risk of bias was assessed using RoB 2.0 and ROBINS-I, and Hedges’ g was pooled using random-effects models. Fourteen studies involving 521 participants aged 3–15 years across seven countries were included; 481 participants were children with autism spectrum disorder and 40 were typically developing comparison participants. Twelve studies used a control group and formed the basis of the primary analysis. Art-based interventions were associated with a small-to-moderate improvement in EI-related social–emotional outcomes (all studies: *k* = 14, SMD = 0.47, 95% CI [0.30, 0.63], *p* < 0.001; controlled studies only: *k* = 12, SMD = 0.46, 95% CI [0.28, 0.64]), with no detectable statistical heterogeneity [*I*^2^ = 0, 95% prediction interval (0.28, 0.65)]. Exploratory subgroup analyses showed no material difference between multimodal and drawing-focused programs (*p* = 0.79) and a larger point estimate for interventions lasting 9–16 weeks, but these moderator findings were descriptive and should be interpreted cautiously. Under the GRADE approach the certainty of the evidence was rated low. Neuroimaging evidence suggests that visual art engagement may support EI through frontoparietal attentional control, graded affective exposure, and prefrontal regulation of limbic reactivity. Direct longitudinal neuroimaging trials are needed to test these proposed mechanisms.

## 1 Introduction

Autism spectrum disorder (ASD) is a neurodevelopmental disorder that affects approximately 1% of the world’s population (Zeidan et al., 2022). It is also associated with social–emotional difficulties, including social–emotional agnosia and alexithymia, which describe challenges in perceiving, understanding, and regulating one’s own emotions and the emotions of others (Beck et al., 2020; McDonald et al., 2024). The amygdala, prefrontal structures, and atypical broader brain activation patterns contribute to these emotional deficits (Greaves, 2025). Deficits in face processing, emotion recognition, and executive functioning lead to poor processing of emotional cues (Hajri et al., 2022). These emotional-intelligence deficits affect nonverbal social communication and add to the challenges faced by children on the spectrum (Keating and Cook, 2020).

Art-based therapy has gained traction in recent years as a therapeutic approach for children with ASD because it enables emotional communication without relying on the verbal strategies required by many traditional therapies (López-Escribano and Orío-Aparicio, 2024). Creating visual art may allow children with ASD to draw on relative visual–spatial processing strengths (Bernier and Li, 2021). Meta-analytic research has shown that visual art therapy can improve psychological well-being in broader clinical populations (Joschko et al., 2024). Parent–child art activities may also support the transfer of therapy gains to everyday home contexts (Moo and Ho, 2025). Reviews of broader creative and multidisciplinary programs, including dramatherapy (Bololia et al., 2022), theater-based social-skills interventions (Martí-Vilar et al., 2023), interactive art activity programs (İnci and Sağlam, 2025), and multidisciplinary art and sport-based interventions (Adıgüzel Akman, 2026), report social–emotional gains but combine visual art with drama, music, or movement; the present review is limited to interventions in which visual art production is the central component, and these broader programs are considered as context rather than pooled in the quantitative synthesis.

Despite the growing evidence base, research findings are limited by small sample sizes, inconsistent protocols, varied outcome measures, and divergent treatment strategies (Chung et al., 2024). No previous meta-analysis has specifically quantified visual art intervention effects on EI-related social–emotional outcomes in children with ASD, and the neurodevelopmental processes through which art activities may improve emotional processing remain unclear (Bradshaw et al., 2022).

The present study addresses these limitations through two primary objectives: conducting a systematic review with meta-analysis to quantify the effects of art-based interventions on EI-related social–emotional outcomes in children with ASD, and synthesizing neuroimaging evidence to construct a neurodevelopmental mechanism framework (Yeung, 2022). By bridging behavioral intervention research and developmental neuroscience, this work generates testable hypotheses about how creative arts therapies may be associated with change in emotion regulation circuitry among children with ASD (Zhang et al., 2021).

## 2 Methods

### 2.1 Search strategy and study selection

This systematic review and meta-analysis was conducted and reported in accordance with the Preferred Reporting Items for Systematic Reviews and Meta-Analyses (PRISMA) 2020 statement. A review protocol defining the research question, eligibility criteria, search strategy, data-extraction procedures and the planned statistical analyses was established *a priori*, before the literature search was executed, and was adhered to throughout; the protocol is provided as Supplementary material, and any deviations from it are identified below. The review was not registered in a public prospective registry such as PROSPERO; this is stated here in accordance with item 24a of the PRISMA 2020 statement, and the completed PRISMA 2020 checklist is provided as Supplementary material. Electronic searches of PubMed, Web of Science, PsycINFO and the Cochrane Library were performed from database inception to 31 December 2025 as the primary search, and were then re-run in full as an update on 15 May 2026, immediately prior to submission, to capture eligible records published after the primary search date; this update yielded one additional eligible study (Yao et al., 2026). During revision, a supplementary targeted update of publicly accessible sources (PubMed/PMC, ClinicalTrials.gov, BioMCP/Europe PMC, and open-web searches) was completed on 17 August 2026 to capture records newly indexed or publicly accessible during the revision period; this update identified one further eligible controlled pilot study (Zhang et al., 2026), a record that was publicly available around the submission period but was captured in this targeted public-source update because of database-indexing and retrieval lag. The search combined three concept blocks: population (“autism” or “ASD”), intervention (“art therapy,” “visual art,” or “drawing therapy”), and outcome (“emotion” or “emotional intelligence”). The search for gray literature was conducted in ProQuest Dissertations and in ClinicalTrials.gov. The complete search strings for each database are provided as Supplementary material.

Study selection followed the PICOS structure. Eligibility was restricted to children aged 3 to 18 years with a clinical diagnosis of ASD. Interventions were required to be visual art-based and to last at least 4 weeks (Schweizer et al., 2022). Eligible comparators included treatment as usual, waitlist, no-treatment controls, typically developing comparison groups, or (where controlled evidence was unavailable) within-group pre and post-intervention comparisons. Controlled designs were prioritized, and uncontrolled pre-post studies were admitted only where controlled evidence was unavailable, because randomization is often infeasible in this population and a controlled-only criterion would have left too few studies for synthesis. Studies using typically developing comparison groups were retained but treated as a source of design heterogeneity, and each included intervention was required to have visual art production as its central active component. Reports without a control group were excluded at full-text review when they did not provide the pre-intervention and post-intervention statistics required to compute a within-group effect size; the two single-arm studies that were retained (Schweizer et al., 2022; Hassan et al., 2025) did provide these statistics and are treated throughout as supportive rather than primary evidence. Programs combining visual art with other modalities were eligible only where the production of visual images was the central activity, which was the case for the art-based somatosensory game (Huili et al., 2023) and the multi-activity art programs (Epp, 2008; Hassan et al., 2025); reports in which the art component could not be separated from music, drama or movement were excluded at full text (*n* = 11). One report that met the remaining criteria was excluded at full text because it provided no pre to post-intervention outcome data: salivary oxytocin was sampled immediately before and immediately after each individual workshop session and expressed as an acute within-session ratio, no concurrent control condition was included, and the authors state that no psychological, emotional or behavioral measure was obtained (Tanaka et al., 2020). This exclusion was made during revision, after the study weights and extracted data were re-checked in response to the Reviewers’ comments, and is reported here as an amendment to the *a priori* protocol in accordance with item 24c of the PRISMA 2020 statement. Katagiri (2009) is the least typical of the included studies because its emotional-understanding task was delivered with a musical component, and its eligibility is acknowledged here as borderline; a sensitivity analysis excluding this study is reported in Section 3.3. The eligibility criteria are presented in Table 1.

**Table 1:** Study eligibility criteria according to PICOS framework.

PICOS element	Inclusion criteria	Exclusion criteria
Population	Children and adolescents aged 3–18 years with clinical ASD diagnosis (DSM-IV, DSM-5, or ICD-10); IQ not specified as exclusion criterion	Participants with severe comorbid neurological conditions (e.g., epilepsy requiring frequent medication adjustment); studies exclusively targeting adults
Intervention	Visual art-based interventions including drawing, painting, sculpture, collage, or multimodal visual arts; minimum duration of 4 weeks	Music therapy alone, drama therapy alone, dance/movement therapy, or combined interventions in which the visual art component cannot be isolated or is not the central activity
Comparison	Treatment-as-usual, waitlist control, no intervention, typically developing comparison groups, or pre-post comparison where controlled designs were unavailable	Studies comparing two active art-based treatments without a control condition
Outcomes	Emotional intelligence or component constructs measured using validated instruments; performance-based assessments, self-report measures, or informant-report instruments accepted	Non-validated measures; behavioral observations without standardized coding
Study Design	Randomized controlled trials or quasi-experimental designs with pre-post assessment; single-arm pre-post designs eligible only where controlled evidence was unavailable and pre and post-intervention statistics were reported	Case reports (*n* < 5), qualitative research, studies with insufficient statistical data for effect size calculation, and uncontrolled studies that did not report the statistics needed to compute a within-group effect size

The eligibility framework in Table 1 was designed to balance practical feasibility against methodological rigor. Because randomization is frequently not feasible in intervention research with pediatric ASD populations, quasi-experimental designs were admitted alongside randomized trials; restricting eligibility to randomized trials alone would have left too few studies for quantitative synthesis. This decision broadens the evidence base at the cost of an increased risk of confounding, which is addressed through the risk-of-bias sensitivity analyses reported in Section 3.3.

Because the included studies used validated instruments that measured EI-related component constructs rather than a single uniform emotional-intelligence battery, the primary outcome was defined as the broad social–emotional domain rather than emotional intelligence narrowly defined, and outcomes were organized into coherent categories for synthesis: emotion recognition and understanding; emotion regulation; broader social–emotional and autism-related functioning; parent–child and family outcomes; and supportive neurobiological indicators. Emotion-recognition and emotion-understanding outcomes were represented by observational or task-based measures of affect identification, art-based emotional expression, and self-representation, including OAT, SPD, IOS, and emotion-understanding measures. Emotion-regulation outcomes were represented by ERC, mYPAS, PWI, and anxiety or affective-adjustment measures. Instruments such as mYPAS were mapped to the emotion-regulation domain as indices of affective adjustment, and peripheral biomarkers such as salivary oxytocin were treated as supportive neurobiological indicators of social-affective processing rather than standalone measures of the primary outcome; after the exclusion described above, no included study contributed an outcome to this category. Social–emotional behavior outcomes were represented by SRS, GARS-2, ATEC, CPRS, and related informant-report indices because these scales capture social reciprocity, adaptive affective behavior, parent–child emotional interaction, and autism-related social–emotional functioning.

### 2.2 Quality assessment and statistical analysis

Two different bias assessment frameworks on randomized and non-randomized studies were used: Cochrane Risk of Bias 2.0 (RoB 2.0) and Risk of Bias in Non-randomized Studies of Interventions (ROBINS-I) for quasi-experimental studies. Each assessment was performed by a single reviewer (LH), and the absence of duplicate independent screening, extraction, and assessment is acknowledged as a major limitation. Standardized forms were used for both risk-of-bias assessment and data extraction, capturing study and participant characteristics, intervention details (art modality and duration), outcome measures, and the statistics needed to compute effect sizes. Where means and standard deviations were reported, effect sizes were computed directly; where studies reported other statistics instead (t, F, η^2^), established conversion formulas were applied (Borenstein et al., 2021).

The primary outcome was the pooled effect on the broad social–emotional domain, expressed as a standardized mean difference; the secondary outcomes were the estimates for the individual outcome domains and for the pre-specified subgroups. Meta-analysis employed random-effects modeling with DerSimonian-Laird estimation using Review Manager 5.4.1 and Stata 17.0, calculating standardized mean differences (Hedges’ g with small-sample correction) and 95% confidence intervals. For controlled studies, effect sizes were based on between-group post-intervention comparisons; when a study reported several eligible measures, a single primary outcome was selected according to a pre-specified hierarchy prioritizing emotion-recognition and emotion-regulation measures, the assessment closest to the end of the intervention was used when multiple time points were available, all scales were oriented so that higher scores denote better functioning, and one effect size per sample was retained to avoid double-counting. For the two single-arm studies, the effect size was the standardized within-group pre-post change, calculated with an assumed pre-post correlation of *r* = 0.50 because the primary reports did not provide it; values of *r* = 0.30 and *r* = 0.70 were examined in sensitivity analysis. Unequal allocation was taken from the primary report where reported (Li et al., 2025, 20 versus 40 participants), and equal group sizes were assumed otherwise; the sensitivity of the standard errors to this assumption was examined under 3:2 and 2:1 allocation ratios. The statistics extracted from each report, the outcome selected, and the resulting Hedges’ g, standard error and study weight are provided in Supplementary Table S4. Statistical heterogeneity was quantified using Q, *I*^2^ and τ^2^, and a 95% prediction interval was calculated; because *I*^2^ is estimated imprecisely when few studies are available, the prediction interval rather than *I*^2^ was used as the primary description of dispersion. Pre-specified subgroup analyses examined intervention modality (drawing-focused versus multimodal visual arts) and duration (≤8 weeks, 9–16 weeks, >16 weeks) as potential moderators. Estimates were also computed separately by study design (randomized controlled trials, non-randomized controlled studies, single-arm pre-post studies, and controlled studies restricted to samples of children with autism spectrum disorder) and, where at least three studies contributed, by outcome domain. Sensitivity analyses included leave-one-out removal, exclusion of studies at serious risk of bias, restriction to the randomized trials, exclusion of the single study with a musical component (Katagiri, 2009), and variation of the assumed pre-post correlation. Publication bias was assessed through funnel plot inspection and Egger’s regression test; with only 14 studies these tests are underpowered, so the absence of strong evidence for small-study effects should not be interpreted as evidence of their absence. The certainty of evidence for the primary outcome was assessed using the GRADE approach, considering risk of bias, inconsistency, indirectness, imprecision, and publication bias.

### 2.3 Neuroimaging evidence synthesis

To address the neurodevelopmental mechanism objective, a complementary narrative synthesis of neuroimaging literature was conducted, as direct meta-analysis of neural outcomes from art-based intervention trials was precluded by the absence of such studies in pediatric ASD populations. Three categories of neuroimaging evidence were systematically reviewed: studies characterizing neural correlates of emotional processing deficits in ASD, investigations examining neural responses during artistic engagement across clinical and neurotypical populations, and research documenting intervention-induced neuroplasticity in ASD. The developmental trajectory of facial emotion processing demonstrates distinct neural specialization patterns in children with ASD compared to neurotypical peers (Golarai et al., 2006). This integrative approach enabled construction of a mechanistic framework connecting observed behavioral outcomes to putative neurodevelopmental pathways, while acknowledging that direct empirical validation remains *a priori*ty for future longitudinal neuroimaging research.

## 3 Results

### 3.1 Literature selection and study characteristics

Following the four-phase PRISMA framework, electronic searches across PubMed, Web of Science, PsycINFO, and Cochrane Library yielded 1,156 records, supplemented by 18 additional records from reference screening and gray literature. After removal of 287 duplicates, 887 records were screened at title and abstract, of which 820 were excluded. Sixty-seven reports were sought for retrieval, all of which were obtained, and 67 were assessed in full text. Fifty-four reports were excluded with reasons, comprising 21 with no control group and insufficient data, 14 with insufficient statistical data for effect-size calculation, 11 in which the art component could not be isolated from music or drama, 7 reporting no EI-related outcome, and 1 (Tanaka et al., 2020) providing only an acute within-session biomarker ratio. Thirteen studies were therefore included after the full database search, and the supplementary public-source update completed on 17 August 2026 identified one further eligible study, giving 14 studies that met the inclusion criteria. The complete selection process is documented in Figure 1.

**Figure 1:**
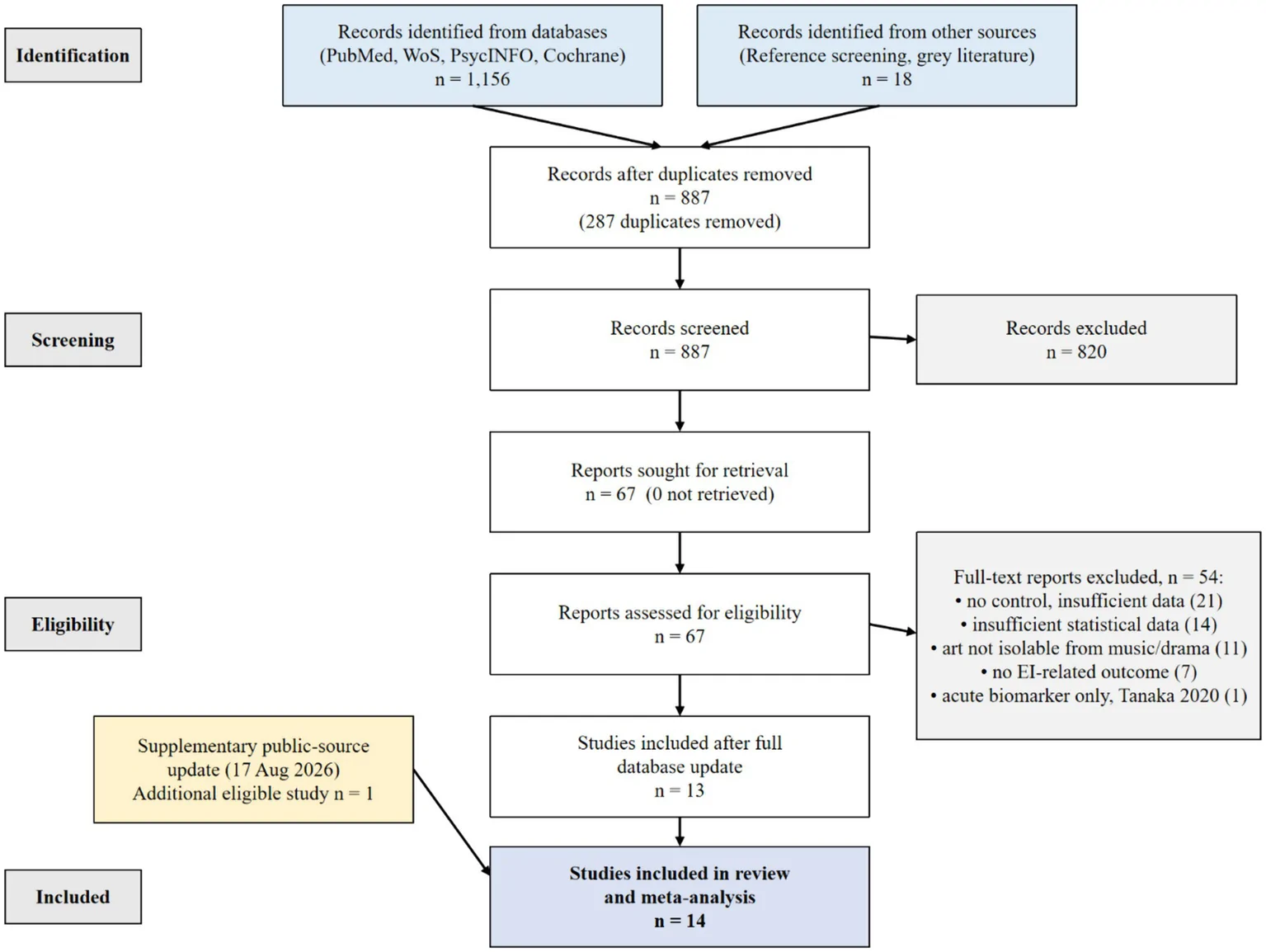
PRISMA flow diagram showing record identification, duplicate removal, title-abstract screening, full-text assessment, exclusions with reasons, and studies included in the qualitative synthesis and meta-analysis, including the 13 studies identified by the full database search and the one further eligible study identified by the supplementary public-source update completed on 17 August 2026.

The 14 included studies comprised 3 RCTs, 9 non-randomized controlled or quasi-experimental studies and 2 single-arm pre-post designs, enrolling 521 participants aged 3–15 years across seven countries, of whom 481 were children with autism spectrum disorder and 40 were typically developing comparison participants (Li et al., 2025). Publication dates ranged from 2008 to 2026. Interventions included family art therapy, drawing programs, somatosensory platforms, and multimodal approaches, with durations of 4–24 weeks. Study characteristics are presented in Table 2.

**Table 2:** Characteristics of studies included in meta-analysis.

Study year	Country	Design	N	Age (years)	Intervention type	Duration (weeks)	Control	EI outcome measure	Risk of bias
Epp, 2008	USA	Quasi	25	6–12	Multimodal	10	TAU	Social skills rating	Moderate
Katagiri, 2009	Japan	Quasi	12	8–15	Multimodal	8	NCC/CC	Emotion understanding	Moderate
Moghaddam et al., 2016	Iran	Quasi	20	5–12	Family art therapy	15	WL	GARS-2	Moderate
Jalambadani, 2020	Iran	Quasi	30	4–10	Drawing	8	WL	SRS	Moderate
Liu et al., 2021	China	RCT	48	3–6	Parent–child art	12	TAU	ATEC	Low
Gómez-Carlier and Powell, 2022	UAE	Non-randomized controlled (pilot)	8	4–10	Online art therapy	8	WL	ERC	Serious
Schweizer et al., 2022	Netherlands	Single-arm pre-post	12	6–12	IOS Art therapy	15	None (single-arm)	OAT	Serious
Huili et al., 2023	China	RCT	26	4–10	Somatosensory game	6	Picture Book	Multiple	Low
An, 2026	China	Quasi	40	3–6	Drawing	24	NI	SRS	Moderate
Hassan et al., 2025	Malaysia	Single-arm pre-post	18	5–12	ART’UISM program	8	None (single-arm)	Multiple	Serious
Li et al., 2025	China	Quasi	60 (20 ASD, 40 TD)	6–12	Drawing	9	TD	fNIRS/SPD	Moderate
Lin et al., 2025	China	RCT	68	3–8	WeChat PCCAT	12	TAU	CPRS/PWI	Low
Yao et al., 2026	China	Quasi	131	3–8	Drawing	8	TAU	mYPAS	Moderate
Zhang et al., 2026	China	Quasi	23	4–7	Mandala drawing	4	TAU	ATEC	Serious

RCT = randomized controlled trial; Quasi = quasi-experimental design; TAU = treatment as usual; WL = waitlist control; NI = no intervention; TD = typically developing children comparison; NCC = no contact control; CC = contact control; Pre-post = pre-post design without separate control group; GARS-2 = Gilliam Autism Rating Scale-2; ERC = Emotion Regulation Checklist; OAT = Observation of a child with Autism in Art Therapy; SRS = Social Responsiveness Scale; SPD = Self-Portrait Drawing; ATEC = Autism Treatment Evaluation Checklist; CPRS = Child–Parent Relationship Scale; PWI = Psychological Well-being Index; mYPAS = modified Yale Preoperative Anxiety Scale; IOS = Images of Self; PCCAT = Parent–Child Creative Art Therapy. Risk of bias assessed using RoB 2.0 for RCTs and ROBINS-I for quasi-experimental studies. Design categories in this table follow the classification used in Section 3.1 and Supplementary Table S4: three RCTs, nine non-randomized or quasi-experimental controlled studies, and two single-arm pre-post designs.

As presented in Table 2, the included studies spanned seven countries; six employed multimodal visual arts programs and eight employed drawing interventions. The outcome set should be interpreted as a synthesis of emotional intelligence and EI-related component constructs, including emotion recognition and understanding, emotion regulation, social responsiveness, parent–child emotional interaction, and affective adjustment.

### 3.2 Methodological quality assessment

Risk of bias evaluation revealed heterogeneous methodological quality reflecting the practical challenges of conducting controlled intervention research in pediatric ASD populations. All three randomized controlled trials assessed using RoB 2.0 demonstrated low overall risk with adequate randomization procedures (Huili et al., 2023; Lin et al., 2025; Liu et al., 2021). Among the eleven non-randomized studies evaluated with ROBINS-I (the nine non-randomized controlled studies together with the two single-arm pre-post designs), seven were judged at moderate risk (Epp, 2008; Katagiri, 2009; Moghaddam et al., 2016; Jalambadani, 2020; An, 2026; Li et al., 2025; Yao et al., 2026), reflecting appropriate baseline adjustment and standardized outcome assessment, and four at serious risk (Gómez-Carlier and Powell, 2022; Schweizer et al., 2022; Hassan et al., 2025; Zhang et al., 2026), primarily because of pre-post designs without concurrent controls, very small samples, class-level allocation, or non-blinded outcome assessment. Blinding of outcome assessors proved challenging given the observable nature of art interventions, although several studies employed independent raters to minimize detection bias. Domain-level RoB 2 and ROBINS-I judgments, with brief justifications for each study, are provided as Supplementary material.

### 3.3 Meta-analysis results

A random-effects meta-analysis was conducted to estimate pooled standardized mean differences and explore sources of between-study variation. Effect sizes were expressed as Hedges’ g with small-sample correction. When studies reported means and standard deviations, effect sizes were calculated directly; when other statistics were reported, established conversion formulas were used as described in Section 2.2. Outcomes were synthesized across operationalizations of EI and related social–emotional constructs, including emotion recognition, emotion regulation, social–emotional behavior and affective adjustment. Figure 2 presents individual study effect sizes with 95% confidence intervals, the pooled estimate, and heterogeneity statistics.

**Figure 2:**
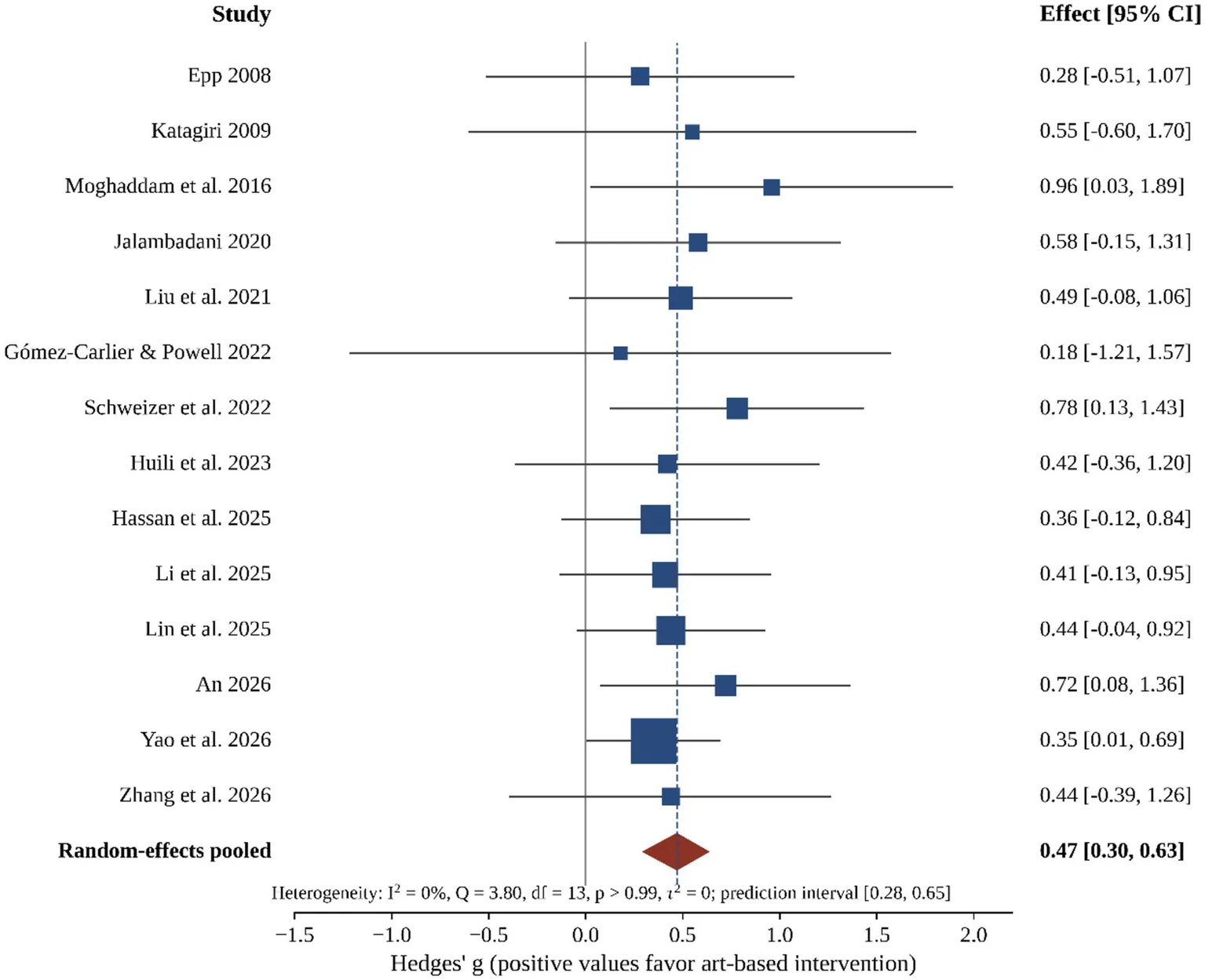
Forest plot of the effects of art-based interventions on EI-related social–emotional outcomes in children with autism spectrum disorder. Squares represent study-level effect sizes (Hedges’ *g*), with square size proportional to the inverse-variance weight assigned to each study; horizontal lines denote 95% confidence intervals. The diamond represents the random-effects pooled estimate [*SMD* = 0.47, 95% *CI* (0.30, 0.63), *p* < 0.001]. Heterogeneity: *I*^2^ = 0%, *Q* = 3.80, *df* = 13, *p* > 0.99, τ^2^ = 0; 95% prediction interval [0.28, 0.65]. Standard errors were recalculated from the reported group sizes during revision and the figure has been redrawn accordingly.

Effect sizes for each individual study, as shown in Figure 2, varied between 0.18 and 0.96. Ten of the fourteen studies had confidence intervals that crossed zero, reflecting the small samples of the individual studies. Study weights, effect-size variances and extracted data were re-checked during revision and the standard errors were recalculated from the reported group sizes; the estimates below reflect the corrected values (the recalculated study-level standard errors, and the effect of each correction stage on the pooled result, are given in Supplementary Tables S6a, and S6b). Across all 14 studies the pooled estimate indicated a statistically significant improvement favoring the art-based interventions (SMD = 0.47, 95% CI [0.30, 0.63], *p* < 0.001), in the range of a small to medium effect, with no detectable statistical heterogeneity (*I*^2^ = 0%, *Q* = 3.80, df = 13, *p* > 0.99, *τ*^2^ = 0, 95% prediction interval [0.28, 0.65]). Because Q was smaller than its degrees of freedom, the observed dispersion of effect sizes was no greater than sampling error alone would produce; with 14 small studies the between-study variance is estimated very imprecisely, so the prediction interval rather than *I*^2^ should be used to judge how much the underlying effect may vary.

Because controlled and uncontrolled designs estimate different contrasts, estimates were also computed separately by design. The 12 controlled studies, which form the primary analysis, yielded SMD = 0.46 (95% CI [0.28, 0.64], *I*^2^ = 0%); restricting these to samples of children with autism spectrum disorder gave SMD = 0.46 (*k* = 11, 95% CI [0.27, 0.65]). The three randomized controlled trials (SMD = 0.45, 95% CI [0.12, 0.79]) and the nine non-randomized controlled studies (SMD = 0.46, 95% CI [0.24, 0.67]) were closely similar, and the two single-arm pre-post studies, presented as supportive rather than primary evidence, gave a slightly larger estimate [SMD = 0.51, 95% CI (0.12, 0.89)]; the estimates for every design-based analysis set are collated in Supplementary Table S6c. Only one study used a typically developing comparison group rather than a no-treatment or treatment-as-usual control [Li et al., 2025, SMD = 0.41, 95% CI (−0.13, 0.95)]; because this design estimates a different contrast it is reported separately and is excluded from the ASD-only estimate above. Estimates by outcome domain were computed where at least three studies contributed: emotion recognition and understanding [*k* = 3, SMD = 0.56, 95% CI (0.17, 0.95)] and broader social–emotional and autism-related functioning [*k* = 8, SMD = 0.50, 95% CI (0.27, 0.73)]. The emotion-regulation (*k* = 2) and parent–child (*k* = 1) domains contained too few studies to support a pooled estimate and are reported descriptively; no included study contributed a neurobiological outcome. All domain-level and design-level estimates are exploratory.

Subgroup analyses examined intervention type and duration. Multimodal visual arts interventions and drawing-focused interventions did not differ materially (multimodal: Epp, 2008; Katagiri, 2009; Moghaddam et al., 2016; Gómez-Carlier and Powell, 2022; Huili et al., 2023; Hassan et al., 2025; drawing-focused: the remaining eight studies) (*k* = 6, SMD = 0.43, 95% CI [0.12, 0.75] vs. *k* = 8, SMD = 0.48, 95% CI [0.29, 0.67]; between-subgroup *χ*^2^ = 0.07, *p* = 0.79), and an alternative classification that assigned the two parent–child programs to the multimodal category gave essentially the same result (*k* = 8, SMD = 0.44 vs. *k* = 6, SMD = 0.49; *p* = 0.79); both modality classifications are reported in Supplementary Table S6e. Duration-based analyses showed a descriptive pattern in which 9–16 week interventions had a larger point estimate than interventions of 8 weeks or less (*k* = 6, SMD = 0.52, 95% CI [0.27, 0.77] vs. *k* = 7, SMD = 0.39, 95% CI [0.16, 0.62]), although the confidence intervals overlapped substantially. The full duration subgroup analysis is given in Supplementary Table S6d. One study delivered an intervention longer than 16 weeks [24 weeks; An, 2026; SMD = 0.72, 95% CI (0.08, 1.36)], so this category is reported for completeness only. Because several subgroup cells contained few studies and the moderator analyses were not statistically significant, these subgroup findings should be interpreted as exploratory rather than confirmatory.

Sensitivity analyses supported the robustness of the pooled estimates. Statistical significance was maintained throughout the leave-one-out analysis, with pooled effect sizes ranging from 0.44 (omitting Schweizer et al., 2022) to 0.50 (95% CI [0.31, 0.69]; omitting Yao et al., 2026, the most heavily weighted study). Excluding the four studies at serious risk of bias left the estimate essentially unchanged (SMD = 0.46, 95% CI [0.28, 0.65], *I*^2^ = 0%, *p* < 0.001) (Gómez-Carlier and Powell, 2022; Hassan et al., 2025; Schweizer et al., 2022; Zhang et al., 2026), as did restriction to the three randomized controlled trials (SMD = 0.45, 95% CI [0.12, 0.79]) and exclusion of the single study with a musical component [*k* = 13, SMD = 0.46, 95% CI (0.30, 0.63); Supplementary Table S6f, which also reports this exclusion across the controlled and randomized analysis sets]. Varying the assumed pre-post correlation for the two single-arm studies between *r* = 0.30 and *r* = 0.70 changed the pooled estimate by less than 0.02. Recomputing the standard errors under 3:2 and 2:1 allocation ratios, rather than the equal group sizes assumed where the primary report did not state them, changed each standard error by less than 0.02 and left the pooled estimate unchanged (Supplementary Table S6g). Funnel plot inspection suggested approximate symmetry, and Egger’s test was non-significant (intercept = 0.55, SE = 0.42, *p* = 0.21), providing no strong evidence of small-study effects; as noted in Section 2.2, this test is underpowered with 14 studies.

### 3.4 Neuroimaging evidence synthesis

A synthesis of the neuroimaging literature identified three domains with convergent evidence connected to possible mechanisms underlying the effects of art-based interventions on children with ASD. Regarding deficits in emotional processing, functional neuroimaging studies of youth with ASD have reported convergent findings: dysfunction of social-brain regions (the posterior superior temporal sulcus and fusiform gyrus); reduced or atypical amygdala responses with poor habituation; and reduced amygdala–ventromedial prefrontal connectivity during the processing of affective faces (Golarai et al., 2006; Dichter, 2012; Swartz et al., 2013; Guo et al., 2024). Studies that have analyzed the neural correlates of artistic engagement in the general population found that the production of drawings and visual arts activated distributed neural networks in the prefrontal executive, cingulate, and the fronto-parietal regions (Bolwerk et al., 2014; Barnett and Vasiu, 2024). In addition, the production of visual arts has been shown to improve the functional connectivity of the default mode network (Bolwerk et al., 2014). Studies of therapeutic interventions in ASD have suggested that some structured programs may be associated with change in the circuits involved in emotion processing, although only one included study acquired neural data during an art-based intervention in children with ASD (Li et al., 2025, functional near-infrared spectroscopy); a further report outside the present synthesis recorded a rise in salivary oxytocin after a participatory art workshop (Tanaka et al., 2020). No included study assessed pre-to-post intervention change in the emotion-processing circuits described above, and none tested neural change as a mediator of behavioral gain. This scarcity precluded quantitative integration of neural with behavioral outcomes and motivates the theoretical mechanisms proposed in the Discussion.

## 4 Discussion

### 4.1 Neural mechanism framework

The meta-analysis indicates a small-to-medium improvement in EI-related social–emotional outcomes among children with ASD (SMD = 0.47 across all studies; SMD = 0.46 when restricted to controlled comparisons). The magnitude is similar to that reported for creative arts therapies in broader populations (Joschko et al., 2024). The absence of detectable heterogeneity (*I*^2^ = 0%) should not be read as evidence that the effect is uniform: with 14 small studies whose effect sizes fall within a narrow range and whose standard errors are comparatively large, the observed dispersion is no greater than sampling error would produce, and the between-study variance cannot be estimated with any precision. The prediction interval reported in the Results gives a more realistic representation of how much the underlying effect may vary.

Neuroimaging research has established that children with ASD exhibit abnormalities in neural circuits subserving emotional processing, particularly involving the amygdala and prefrontal regions (Guo et al., 2024). Functional MRI studies have documented reduced amygdala-prefrontal connectivity during emotional face processing (Swartz et al., 2013). Earlier comprehensive reviews similarly describe hypoactivation of social brain regions together with reduced functional connectivity (Dichter, 2012).

Neural activation has been documented within distributed networks, including prefrontal executive systems, while participants engage in various forms of art production (Barnett and Vasiu, 2024). For instance, prefrontal and cingulate cortices are activated during drawing tasks, and visual art production facilitates the connectivity of the default mode networks (Bolwerk et al., 2014). The point estimates for multimodal and drawing-focused programs did not differ materially in the present data (SMD = 0.43 versus 0.48, *p* = 0.79), so these findings provide no support for the suggestion that multisensory stimulation confers additional benefit. Initial fNIRS data suggest that a drawing intervention was accompanied by prefrontal hemodynamic changes in children with ASD (Li et al., 2025), and a participatory art workshop not included in the present synthesis was followed by a rise in salivary oxytocin in children with ASD (Tanaka et al., 2020).

Beyond neuroimaging in ASD and art-production studies in other populations, a small body of non-art intervention research in children with ASD—including social-skills programs and structured behavioral therapies—has documented pre to post-intervention change in emotion-related neural circuitry, indicating that intervention-related plasticity in these systems is in principle detectable in this age range. Because these studies did not deliver art-based interventions, they cannot support the modality-specific contribution of visual-art production and are treated here only as background evidence for the general feasibility of the pathways proposed below, rather than as direct evidence for any of them.

Drawing together these five strands of evidence—art interventions in ASD, observational neuroimaging in ASD, art-production studies in other populations, non-art intervention studies in ASD, and theoretical inference—the authors propose that art-based interventions may improve EI through three overlapping, non-exclusive pathways. First, the sustained visual–motor demands of drawing and constructing may strengthen recruitment of the frontoparietal network, which supports the attentional control on which emotion recognition and appraisal depend. Second, repeatedly depicting and discussing affective content provides graded, low-arousal exposure to emotional material, which may attenuate amygdala reactivity and improve amygdala habituation under conditions less aversive than direct face-to-face social exchange. Third, the regulatory demands of an extended creative task—tolerating error, revising, and delaying completion—may engage prefrontal control over limbic reactivity and, with repetition, strengthen amygdala–prefrontal coupling. Each pathway is a hypothesis derived from convergent but indirect evidence; none is tested by the studies synthesized here, and all three require longitudinal designs acquiring neural and behavioral outcomes concurrently.

### 4.2 Clinical implications

The descriptive duration pattern has potential clinical implications but, as an exploratory subgroup finding, should be interpreted cautiously. Interventions of 9–16 weeks produced a larger point estimate (SMD = 0.52) than programs of 8 weeks or less (SMD = 0.39), which may indicate that a moderate duration is preferable to a brief one, although the confidence intervals overlapped and the between-subgroup difference was not statistically significant. The single study exceeding 16 weeks yielded a larger estimate still (SMD = 0.72), but with only one study in that subgroup no conclusion can be drawn about longer durations, and the apparent trend should not be read as an established dose–response relationship. The estimate remained significant after excluding methodologically weaker studies (SMD = 0.46, 95% CI [0.28, 0.65]), indicating that it is not driven by the studies at serious risk of bias, although this does not by itself establish a causal effect. These findings are consistent with the use of art-based approaches as one component of multicomponent ASD intervention programs, particularly for children with visual–spatial strengths or difficulty engaging with verbal therapies. Family-centered approaches incorporating parent–child engagement show potential for generalizing gains to home environments (Moghaddam et al., 2016). Reviews of broader creative and multidisciplinary programs (Bololia et al., 2022; Martí-Vilar et al., 2023; İnci and Sağlam, 2025; Adıgüzel Akman, 2026) report comparable social–emotional gains, but because they combine visual art with drama, music or movement they cannot isolate the contribution of visual art production; the present estimate should therefore not be read as a general estimate for creative-arts therapy.

### 4.3 Limitations and future directions

Several limitations warrant consideration. Although the review followed an a priori protocol, that protocol was not deposited in a public prospective registry, and the possibility of *post hoc* deviations therefore cannot be formally excluded, which reduces protection against undisclosed changes and should temper confidence in the findings. The inclusion of a small number of single-arm and typically developing comparison designs also means that part of the pooled estimate reflects within-group change rather than a between-group contrast. Because the variance of a standardized within-group change depends on the single-group sample size, the two single-arm studies also carry more weight in the all-study analysis than their design warrants (together 18.2% of the total weight), which is a further reason the controlled comparisons rather than the all-study estimate are treated as the primary analysis. The predominance of quasi-experimental designs introduces potential confounding that RCTs would better address. Screening, data extraction and risk-of-bias assessment were performed by a single reviewer, which increases the risk of selection and extraction error relative to duplicate independent assessment. Outcome measurement heterogeneity may have obscured intervention effects on specific emotional processing facets. Relatedly, several included studies indexed EI using instruments developed for adjacent constructs—social responsiveness, autism severity, or parent–child relationship quality—rather than dedicated measures of emotional intelligence; the pooled estimate should therefore be interpreted as an effect on a broad social–emotional domain rather than on emotional intelligence narrowly defined. The interventions themselves ranged from drawing and painting to family and online art therapy and multimodal programs, and this clinical heterogeneity, together with the combination of outcomes drawn from different instruments, limits the specificity of any single pooled estimate, so the domain-level estimates reported in Section 3.3 should be regarded as exploratory and no firm domain-specific conclusions can be drawn from the present data. Subgroup analyses were based on small numbers of studies per category and should be considered exploratory. Sensitivity analyses left the direction and approximate magnitude of the pooled estimate unchanged: leave-one-out estimates ranged from 0.44 to 0.50, exclusion of the four studies at serious risk of bias gave 0.46 [95% CI (0.28, 0.65)], restriction to the randomized trials gave 0.45 [95% CI (0.12, 0.79)], and exclusion of the study with a musical component gave 0.46 [95% CI (0.30, 0.63)]. The eligibility of that study (Katagiri, 2009) against the visual-art criterion applied here is acknowledged as borderline. Under the GRADE approach, the certainty of evidence for the primary outcome was rated low, downgraded mainly for risk of bias and imprecision arising from predominantly quasi-experimental designs and small samples. The data extracted for every included study were re-checked against the source reports during revision, and the standard errors were recalculated from the reported group sizes; because extraction was not independently duplicated, minor extraction error cannot be fully excluded, and the sensitivity analyses reported above were used to gauge the robustness of the pooled estimate to any single study. The proposed neurodevelopmental framework remains theoretical: only one included study acquired neural data during an art-based intervention (Li et al., 2025), no included study assessed pre-to-post intervention change in the emotion-processing circuits described, and none tested neural change as a mediator of behavioral gain. Future research priorities include longitudinal neuroimaging studies examining pre-to-post intervention changes in emotion-processing circuits with concurrent behavioral assessment. Additional studies should examine moderators of treatment response, including baseline emotional intelligence, ASD severity, and cognitive ability, to facilitate personalized interventions.

For the field to move from a plausible framework to a tested one, three design features are needed. First, trials should acquire task-based neural outcomes that map onto the proposed circuits, for example an emotional face-processing paradigm indexing amygdala and fusiform responsivity together with a frontoparietal-dependent emotion-regulation task, rather than resting-state measures alone. Second, functional near-infrared spectroscopy is the most feasible modality for young, movement-prone children with ASD and can be acquired during the artistic activity itself, permitting a within-session test of whether prefrontal and inferior parietal recruitment during drawing predicts subsequent gains in emotion recognition. Third, at least three measurement occasions (baseline, mid-intervention and post-intervention, with follow-up) are required to distinguish intervention-related neural change from typical maturation, and this change should be formally tested as a mediator of behavioral change rather than reported in parallel with it. Analysis plans, target sample sizes and candidate mediators for such trials should be registered prospectively.

## 5 Conclusion

This systematic review and meta-analysis, conducted in accordance with PRISMA 2020 guidelines and synthesizing evidence from 14 studies involving 521 participants, provides, to our knowledge, the first quantitative estimate of art-based intervention effects on EI-related social–emotional outcomes in this population. Random-effects meta-analysis revealed a statistically significant small-to-medium effect favoring art-based interventions [all studies: SMD = 0.47, 95% CI (0.30, 0.63), *p* < 0.001; controlled studies only: SMD = 0.46, 95% CI (0.28, 0.64)], with no detectable statistical heterogeneity [*I*^2^ = 0%, *τ*^2^ = 0, 95% prediction interval (0.28, 0.65)]; given the small number of studies, this reflects limited between-study dispersion rather than confirming uniform effects. Subgroup patterns showed no material difference between multimodal and drawing-focused programs and a larger point estimate for treatment durations of 9–16 weeks, but these moderator findings should be interpreted as exploratory because subgroup cells were small and between-subgroup differences were not statistically significant. Under the GRADE approach the certainty of the evidence for the primary outcome was rated low.

The integration of behavioral findings with convergent neuroimaging evidence motivates a mechanistic framework proposing that art-based interventions may support social–emotional functioning through pathways hypothesized to involve frontoparietal attentional control, amygdala–prefrontal regulation of affective reactivity, and experience-dependent change in emotion-processing circuits during visual-motor engagement. Because no included study tested these pathways or examined neural change as a mediator of behavioral improvement, the framework is presented as a set of testable hypotheses rather than an established mechanism. These findings provide preliminary support for considering art-based approaches as an adjunctive non-verbal component of multicomponent programs that capitalize on the visual–spatial processing strengths characteristic of this population, rather than as an established treatment. Because the certainty of the evidence remains limited, future longitudinal neuroimaging research is needed to directly test the proposed neurodevelopmental mechanisms linking artistic engagement to EI-related social–emotional gains.

## Data Availability

The original contributions presented in the study are included in the article/Supplementary material, further inquiries can be directed to the corresponding author.
