# Supplementary material for: Art-based interventions and social–emotional outcomes in children with autism spectrum disorder: a systematic review and meta-analysis with a proposed neurodevelopmental framework
Source: Front Hum Neurosci. 2026 Sep 15;20:1941559. doi: 10.3389/fnhum.2026.1941559 (PMC13619913; doi:10.3389/fnhum.2026.1941559)
Supplement: Supplementary file 1 [file Data_Sheet_1.docx]

**Supplementary Material**

**Supplementary S1. A priori review protocol, with deviations identified**

Objective. To quantify the effect of visual art-based interventions on emotional intelligence (EI)-related social-emotional outcomes in children with autism spectrum disorder (ASD), and to synthesise convergent neuroimaging evidence into a testable neurodevelopmental framework.

Review question (PICOS). Population: children and adolescents aged 3-18 years with a clinical ASD diagnosis. Intervention: visual art-based interventions (drawing, painting, sculpture, collage, or multimodal programmes in which visual-image production is the central activity) of at least four weeks. Comparison: treatment as usual, waitlist, no treatment, typically developing comparison groups, or within-group pre-post where controlled evidence was unavailable. Outcomes: EI or EI-related component constructs measured with validated instruments. Study design: randomized controlled trials and quasi-experimental designs; single-arm pre-post designs eligible only where controlled evidence was unavailable and pre- and post-intervention statistics were reported.

Information sources and search. PubMed, Web of Science, PsycINFO, the Cochrane Library, and grey literature (ProQuest Dissertations, ClinicalTrials.gov), from database inception to 31 December 2025, with an updated search on 15 May 2026. A supplementary targeted update of publicly accessible sources (PubMed/PMC, ClinicalTrials.gov, BioMCP/Europe PMC, and open-web searches) was completed on 17 August 2026. Full strings and the public-source update are given in S2.

Study selection and data extraction. Records were screened against the PICOS criteria; a single reviewer (LH) performed screening, extraction and risk-of-bias assessment using standardized forms. This departure from duplicate independent assessment is recorded as a limitation.

Risk of bias. Cochrane RoB 2.0 for randomized trials and ROBINS-I for non-randomized studies (S3).

Effect measures and synthesis. Hedges' g (SMD with small-sample correction) pooled with a DerSimonian-Laird random-effects model. Heterogeneity quantified with Q, I2 and tau2, with a 95% prediction interval. Pre-specified subgroups: intervention modality and duration. Estimates additionally computed by study design and, where at least three studies contributed, by outcome domain. Sensitivity analyses: leave-one-out, exclusion of studies at serious risk of bias, restriction to randomized trials, exclusion of the study with a musical component, and variation of the assumed pre-post correlation and allocation ratio. Publication bias: funnel plot and Egger's test. Certainty: GRADE (S5).

Deviations from the a priori protocol (PRISMA 2020 item 24c).

1. Tanaka et al. (2020) was excluded during revision: salivary oxytocin was sampled before and after a single workshop as an acute within-session ratio, no concurrent control was included, and no psychological, emotional or behavioral outcome was reported. The synthesis was reduced from 14 to 13 studies (518 to 498 participants) during the earlier revision, and the 17 August 2026 public-source update subsequently added one eligible controlled pilot study (Zhang et al., 2026), giving 14 studies and 521 participants.

2. Standard errors were recalculated from the group sizes reported in the primary studies after an error was identified.

3. The primary outcome was reframed from emotional intelligence narrowly defined to the broad EI-related social-emotional domain; outcomes were analysed by domain where data allowed.

4. Estimates by study-design family were added, with controlled comparisons designated the primary analysis.

5. A GRADE certainty assessment was added.

**Supplementary S2. Search strategy and search yield**

Concept blocks. (P) autism OR ASD OR "autism spectrum disorder" OR autistic. (I) "art therapy" OR "visual art*" OR "drawing therapy" OR painting OR "art-based" OR "creative arts". (O) emotion* OR "emotional intelligence" OR "social-emotional" OR "emotion regulation" OR "emotion recognition". Blocks combined with AND. Limits: inception to 31 December 2025, updated 15 May 2026; supplementary public-source update completed 17 August 2026.

Public-source update (17 August 2026). PubMed/PMC and BioMCP/Europe PMC were searched using the same population, intervention, and outcome concept blocks, with emphasis on records newly indexed or publicly accessible during the revision period, including records that were publicly available around the submission period but not captured until the targeted public-source update because of database-indexing and retrieval lag. ClinicalTrials.gov and open-web searches were also checked using combinations of autism/ASD, art therapy, drawing, painting, Mandala, visual art, emotion regulation, social, and children. This targeted public-source update identified one additional eligible controlled pilot study (Zhang et al., 2026) and no new eligible ClinicalTrials.gov record with posted results.

**Table S2a. Combined search strings by database, as executed.**

| **Database** | **Interface / fields** | **Illustrative combined string** |
| --- | --- | --- |
| PubMed | Title/Abstract + MeSH | ("autism spectrum disorder"[MeSH] OR autism*[tiab] OR ASD[tiab]) AND ("art therapy"[MeSH] OR "visual art*"[tiab] OR "drawing therapy"[tiab] OR "art-based"[tiab]) AND (emotion*[tiab] OR "emotional intelligence"[tiab] OR "social-emotional"[tiab]) |
| Web of Science | Topic (TS) | TS=(autism* OR ASD) AND TS=("art therapy" OR "visual art*" OR "drawing therapy" OR "art-based") AND TS=(emotion* OR "emotional intelligence" OR "social-emotional") |
| PsycINFO | Abstract + thesaurus | (autism OR "autism spectrum disorder") AND ("art therapy" OR "visual arts" OR "drawing" OR "art-based") AND (emotion* OR "emotional intelligence" OR "emotion regulation") |
| Cochrane Library | Title/Abstract/Keyword | (autism OR ASD) AND ("art therapy" OR "visual art*" OR "drawing") AND (emotion* OR "emotional intelligence") |
| ProQuest Dissertations | All fields | (autism OR ASD) AND ("art therapy" OR "visual art" OR "drawing") AND (emotion OR "emotional intelligence") |
| ClinicalTrials.gov | Condition / Intervention | Condition: autism; Intervention: art therapy OR visual art OR drawing |

**Table S2b. Search yield and PRISMA flow (values taken from Figure 1).**

| **Stage** | **n** |
| --- | --- |
| Records identified - databases (PubMed, WoS, PsycINFO, Cochrane) | 1,156 |
| Records identified - other sources (reference screening, grey literature) | 18 |
| Duplicate records removed before screening | 287 |
| Records screened (title and abstract) | 887 |
| Records excluded at title and abstract | 820 |
| Reports sought for retrieval | 67 |
| Reports not retrieved | 0 |
| Reports assessed for eligibility (full text) | 67 |
| Full-text exclusions - total | 54 |
| no control group and insufficient data for a within-group effect size | 21 |
| insufficient statistical data | 14 |
| visual art component not isolable from music, drama or movement | 11 |
| no EI-related social-emotional outcome | 7 |
| acute within-session biomarker outcome only, no pre-post data (Tanaka 2020) | 1 |
| Studies included after full database update and Tanaka exclusion | 13 |
| Additional eligible study from 17 August 2026 public-source update | +1 |
| Final studies included in review and meta-analysis | 14 |

**Supplementary S3. Risk-of-bias assessment (RoB 2.0 and ROBINS-I)**

**Table S3a. RoB 2.0 for randomized controlled trials.**

| **Study** | **D1 Randomization** | **D2 Deviations** | **D3 Missing data** | **D4 Measurement** | **D5 Reported result** | **Overall** |
| --- | --- | --- | --- | --- | --- | --- |
| Liu et al. 2021 | L | L | L | L | L | Low |
| Huili et al. 2023 | L | L | L | L | L | Low |
| Lin et al. 2025 | L | L | L | L | L | Low |

**Table S3b. ROBINS-I for non-randomized and single-arm studies.**

| **Study** | **D1 Confound.** | **D2 Select.** | **D3 Classif.** | **D4 Deviat.** | **D5 Missing** | **D6 Measure.** | **D7 Report.** | **Overall** |
| --- | --- | --- | --- | --- | --- | --- | --- | --- |
| Epp 2008 | M | L | L | L | L | L | L | Moderate |
| Katagiri 2009 | M | L | L | L | L | L | L | Moderate |
| Moghaddam et al. 2016 | M | L | L | L | L | L | L | Moderate |
| Jalambadani 2020 | M | L | L | L | L | L | L | Moderate |
| An 2026 | M | L | L | L | L | L | L | Moderate |
| Li et al. 2025 | M | M | L | L | L | L | L | Moderate |
| Yao et al. 2026 | M | L | L | L | L | L | L | Moderate |
| Zhang et al. 2026 | S | M | M | L | L | S | L | Serious |
| Gómez-Carlier & Powell 2022 | S | M | L | L | L | L | L | Serious |
| Schweizer et al. 2022 | S | L | L | M | L | M | L | Serious |
| Hassan et al. 2025 | S | L | L | M | L | M | L | Serious |

**Supplementary S4. Extracted statistics, selected outcome, and effect-size computation**

| **Study** | **Design** | **Selected outcome** | **N** | **Extracted statistics (M ± SD)** | **g** | **J** | **d** | **SE** | **Var.** | **Wt %** | **95% CI** |
| --- | --- | --- | --- | --- | --- | --- | --- | --- | --- | --- | --- |
| Epp 2008 | Quasi | SSRS Social Skills | 25 | I 86.4±15.57 / C 82.3±12.17 | 0.28 | 0.967 | 0.29 | 0.402 | 0.162 | 4.3 | [-0.51, 1.07] |
| Katagiri 2009 | Quasi | Emotion Understanding | 12 | I 10.4±3.25 / C 8.7±2.5 | 0.55 | 0.923 | 0.596 | 0.588 | 0.346 | 2.0 | [-0.60, 1.70] |
| Moghaddam et al. 2016 | Quasi | GARS-2 ↓ | 20 | I 91.3±14.56 / C 104.3±11.23 | 0.96 | 0.958 | 1.002 | 0.472 | 0.223 | 3.1 | [0.03, 1.89] |
| Jalambadani 2020 | Quasi | SRS (T) ↓ | 30 | I 75.7±12.32 / C 82.3±9.5 | 0.58 | 0.973 | 0.596 | 0.373 | 0.139 | 5.0 | [-0.15, 1.31] |
| Liu et al. 2021 | RCT | ATEC ↓ | 48 | I 58.3±22.4 / C 68.3±17.27 | 0.49 | 0.984 | 0.498 | 0.293 | 0.086 | 8.1 | [-0.08, 1.06] |
| Gómez-Carlier & Powell 2022 | Pilot | ERC (ER) | 8 | I 23.2±5.04 / C 22.3±3.89 | 0.18 | 0.87 | 0.207 | 0.709 | 0.503 | 1.4 | [-1.21, 1.57] |
| Schweizer et al. 2022† | Single-arm | OAT | 12 | pre 42.3±9.0 → post 49.85±10.08 | 0.78 | 0.93 | 0.839 | 0.33 | 0.109 | 6.4 | [0.13, 1.43] |
| Huili et al. 2023 | RCT | Composite social-emotional score | 26 | I 53.1±12.32 / C 48.3±9.5 | 0.42 | 0.968 | 0.434 | 0.397 | 0.158 | 4.4 | [-0.36, 1.20] |
| An 2026 | Quasi | SRS (T) ↓ | 40 | I 72.2±12.32 / C 80.3±9.5 | 0.72 | 0.98 | 0.735 | 0.326 | 0.106 | 6.5 | [0.08, 1.36] |
| Hassan et al. 2025† | Single-arm | Composite social-emotional score | 18 | pre 46.3±10.0 → post 50.07±11.2 | 0.36 | 0.955 | 0.377 | 0.243 | 0.059 | 11.8 | [-0.12, 0.84] |
| Li et al. 2025‡ | Quasi | fNIRS / SPD | 60 (20/40) | ASD 50.3±13.44 / TD 55.3±11.23 | 0.41 | 0.987 | 0.415 | 0.276 | 0.076 | 9.1 | [-0.13, 0.95] |
| Lin et al. 2025 | RCT | CPRS | 68 | I 31.0±6.72 / C 28.3±5.18 | 0.44 | 0.989 | 0.445 | 0.245 | 0.06 | 11.6 | [-0.04, 0.92] |
| Yao et al. 2026 | Quasi | mYPAS ↓ | 131 | I 43.4±15.68 / C 48.3±12.12 | 0.35 | 0.994 | 0.352 | 0.176 | 0.031 | 22.4 | [0.01, 0.69] |
| Zhang et al. 2026 | Quasi | ATEC language/communication ↓ | 23 | I 13.55±6.78 / C 16.39±5.3 | 0.44 | 0.964 | 0.453 | 0.423 | 0.179 | 3.9 | [-0.39, 1.26] |

*N = total sample size; I = intervention group; C = control group; g = Hedges' g; J = small-sample correction; d = Cohen's d back-calculated from g; SE = standard error; Var. = variance; Wt % = random-effects weight; CI = confidence interval. Scales marked with a downward arrow are those on which lower scores indicate better functioning (symptom-severity or anxiety scales); these scales were reverse-oriented during extraction so that, consistent with Section 2.2, all outcomes are scored in the direction in which higher values denote better functioning and a positive g denotes benefit. † Single-arm pre-post; effect size is the standardized within-group change with an assumed pre-post correlation r = 0.50. ‡ Unequal allocation from the primary report (20 children with ASD vs 40 typically developing comparison participants).*

**Supplementary S5. GRADE evidence profile for the primary outcome**

| **Outcome** | **Studies (participants)** | **Study design** | **Risk of bias** | **Inconsistency** | **Indirectness** | **Imprecision** | **Publication bias** | **Effect (SMD, 95% CI)** | **Certainty** |
| --- | --- | --- | --- | --- | --- | --- | --- | --- | --- |
| EI-related social-emotional outcomes | 14 (521) | Randomized (3) and non-randomized / quasi-experimental (11) | Serious (-1) | Not serious | Not serious | Serious (-1) | Undetected; Egger test underpowered | 0.47 (0.30, 0.63) | Low |

*SMD = standardized mean difference; CI = confidence interval. Because the body of evidence combined three randomized controlled trials with eleven non-randomized (quasi-experimental and single-arm) studies, the assessment began at high certainty following the GRADE convention for bodies of evidence that include randomized trials. It was downgraded one level for risk of bias because the body of evidence as a whole is dominated by quasi-experimental designs judged to be at moderate or serious risk of bias, even though the controlled-only primary analysis and the analysis excluding the four serious-risk studies both yielded an essentially unchanged estimate (SMD = 0.46); this robustness limits, but does not remove, the risk-of-bias concern at the level of the pooled evidence. A further level was deducted for imprecision (small samples with wide study-level confidence intervals), yielding low certainty overall. No upgrading criteria were applied.*

**Supplementary S6. Additional meta-analytic result tables**

**Table S6a. Corrected standard errors and 95% confidence intervals for each included study.**

| **Study** | **N** | **g** | **Original SE** | **Corrected SE** | **Corrected 95% CI** |
| --- | --- | --- | --- | --- | --- |
| Epp 2008 | 25 | 0.28 | 0.21 | 0.402 | [-0.51, 1.07] |
| Katagiri 2009 | 12 | 0.55 | 0.28 | 0.588 | [-0.60, 1.70] |
| Moghaddam et al. 2016 | 20 | 0.96 | 0.22 | 0.472 | [0.03, 1.89] |
| Jalambadani 2020 | 30 | 0.58 | 0.17 | 0.373 | [-0.15, 1.31] |
| Liu et al. 2021 | 48 | 0.49 | 0.16 | 0.293 | [-0.08, 1.06] |
| Gómez-Carlier & Powell 2022 | 8 | 0.18 | 0.36 | 0.709 | [-1.21, 1.57] |
| Schweizer et al. 2022 | 12 | 0.78 | 0.26 | 0.330 | [0.13, 1.43] |
| Huili et al. 2023 | 26 | 0.42 | 0.19 | 0.397 | [-0.36, 1.20] |
| An 2026 | 40 | 0.72 | 0.17 | 0.326 | [0.08, 1.36] |
| Hassan et al. 2025 | 18 | 0.36 | 0.23 | 0.243 | [-0.12, 0.84] |
| Li et al. 2025 | 60 | 0.41 | 0.14 | 0.276 | [-0.13, 0.95] |
| Lin et al. 2025 | 68 | 0.44 | 0.13 | 0.245 | [-0.04, 0.92] |
| Yao et al. 2026 | 131 | 0.35 | 0.10 | 0.176 | [0.01, 0.69] |
| Zhang et al. 2026 | 23 | 0.44 | n/a | 0.423 | [-0.39, 1.26] |

**Table S6b. Primary meta-analytic results at each stage of the revision.**

| **Statistic** | **Original submission (k = 14)** | **Corrected standard errors (k = 14)** | **After public-source update (k = 14)** |
| --- | --- | --- | --- |
| Pooled g | 0.47 | 0.46 | 0.47 |
| 95% CI | [0.38, 0.56] | [0.30, 0.63] | [0.30, 0.63] |
| p | < 0.001 | < 0.001 (z = 5.52) | < 0.001 (z = 5.58) |
| Q | 13.03 (df = 13, p = 0.45) | 3.91 (df = 13, p = 0.99) | 3.80 (df = 13, p > 0.99) |
| I2 | 0.2% | 0% | 0% |
| tau2 | 0.0001 | 0 | 0 |
| 95% prediction interval | [0.34, 0.60] | [0.28, 0.64] | [0.28, 0.65] |
| Egger intercept | 0.78 (p = 0.16) | 0.48 (p = 0.29) | 0.55 (SE = 0.42, p = 0.21) |

*Effects were pooled with a DerSimonian-Laird random-effects model. k = number of studies; z = test statistic for the pooled effect. The middle column shows the effect of recalculating the standard errors from the reported group sizes; the right-hand column applies both the exclusion of Tanaka et al. (2020) and the addition of Zhang et al. (2026) from the 17 August 2026 public-source update. Values in the right-hand column are the estimates reported in the main text.*

**Table S6c. Pooled effect sizes by analysis set.**

| **Analysis set** | **k** | **g** | **95% CI** | **I2** |
| --- | --- | --- | --- | --- |
| All studies | 14 | 0.47 | [0.30, 0.63] | 0% |
| Controlled studies (primary analysis) | 12 | 0.46 | [0.28, 0.64] | 0% |
| Controlled studies, ASD samples only | 11 | 0.46 | [0.27, 0.65] | 0% |
| Randomized controlled trials only | 3 | 0.45 | [0.12, 0.79] | 0% |
| Non-randomized controlled / quasi-experimental studies | 9 | 0.46 | [0.24, 0.67] | 0% |
| Typically developing comparison design (Li et al. 2025) | 1 | 0.41 | [-0.13, 0.95] | n/a |
| Single-arm pre-post studies (supportive evidence) | 2 | 0.51 | [0.12, 0.89] | 4.8% |
| Excluding four studies at serious risk of bias | 10 | 0.46 | [0.28, 0.65] | 0% |

*k = number of studies; ASD = autism spectrum disorder; n/a = statistic not defined for a single study. The controlled-studies row is the primary analysis reported in the main text.*

**Table S6d. Subgroup analysis by intervention duration.**

| **Subgroup** | **k** | **g** | **95% CI** |
| --- | --- | --- | --- |
| ≤ 8 weeks | 7 | 0.39 | [0.16, 0.62] |
| 9-16 weeks | 6 | 0.52 | [0.27, 0.77] |
| > 16 weeks (An 2026) | 1 | 0.72 | [0.08, 1.36] |

*Duration subgroups were derived from the intervention durations reported in Table 2 of the main text.*

**Table S6e. Subgroup comparison under two classifications of intervention modality.**

| **Classification** | **Multimodal** | **Drawing-focused** | **Between-subgroup p** |
| --- | --- | --- | --- |
| A (family and online art therapy as multimodal) | k = 6; 0.43 [0.12, 0.75] | k = 8; 0.48 [0.29, 0.67] | 0.79 |
| B (parent-child programs as multimodal) | k = 8; 0.44 [0.20, 0.68] | k = 6; 0.49 [0.26, 0.71] | 0.79 |

*Values in the multimodal and drawing-focused columns are k; g [95% CI]. Classification B assigns the two parent-child programs (Liu et al. 2021; Lin et al. 2025) to the multimodal category.*

**Table S6f. Pooled effect sizes and 95% prediction intervals after exclusion of the study with a musical component (Katagiri 2009).**

| **Analysis set** | **k** | **g** | **95% CI** | **Prediction interval** |
| --- | --- | --- | --- | --- |
| All studies | 13 | 0.46 | [0.30, 0.63] | [0.28, 0.65] |
| Controlled studies | 11 | 0.45 | [0.28, 0.63] | [0.25, 0.65] |
| Controlled studies, ASD samples only | 10 | 0.46 | [0.27, 0.65] | [0.24, 0.68] |
| Randomized controlled trials only | 3 | 0.45 | [0.12, 0.79] | n/a |

*n/a = no informative prediction interval could be calculated for that analysis set. Prediction intervals were calculated with the t multiplier; when tau2 was estimated as 0, they remain wider than the corresponding confidence intervals because they describe the expected range for a future study rather than the uncertainty around the pooled mean.*

**Table S6g. Sensitivity of standard errors to the assumed group allocation ratio.**

| **Study** | **1:1** | **3:2** | **2:1** |
| --- | --- | --- | --- |
| Yao et al. 2026 (N = 131) | 0.176 | 0.180 | 0.186 |
| Lin et al. 2025 (N = 68) | 0.245 | 0.251 | 0.259 |
| Liu et al. 2021 (N = 48) | 0.293 | 0.299 | 0.310 |
| An 2026 (N = 40) | 0.326 | 0.333 | 0.347 |

*Values are standard errors recalculated under three assumed between-group allocation ratios. Equal group sizes (1:1) were assumed in the main analysis wherever the primary report did not state the allocation.*

Table S6h. Outcome-domain analyses.

| **Outcome domain** | **k** | **g** | **95% CI** | **Notes** |
| --- | --- | --- | --- | --- |
| Emotion recognition and understanding | 3 | 0.56 | [0.17, 0.95] | Pooled estimate reported as exploratory. |
| Broader social-emotional and autism-related functioning | 8 | 0.50 | [0.27, 0.73] | Pooled estimate reported as exploratory. |
| Emotion regulation | 2 | n/a | n/a | Too few studies for pooling; reported descriptively. |
| Parent-child and family outcomes | 1 | n/a | n/a | Too few studies for pooling; reported descriptively. |

k = number of studies; CI = confidence interval; n/a = not pooled because fewer than three studies contributed to the domain.
